# Evaluation of *α*-Glucosidase Inhibitory Effect of 50% Ethanolic Standardized Extract of* Orthosiphon stamineus* Benth in Normal and Streptozotocin-Induced Diabetic Rats

**DOI:** 10.1155/2015/754931

**Published:** 2015-11-16

**Authors:** Elsnoussi Ali Mohamed, Mariam Ahmad, Lee Fung Ang, Mohd. Zaini Asmawi, Mun Fei Yam

**Affiliations:** School of Pharmaceutical Sciences, Universiti Sains Malaysia, 11800 Penang, Malaysia

## Abstract

In the present study, a 50% ethanolic extract of* Orthosiphon stamineus* was tested for its *α*-glucosidase inhibitory activity.* In vivo* assays of the extract (containing 1.02%, 3.76%, and 3.03% of 3′hydroxy-5,6,7,4′-tetramethoxyflavone, sinensetin, and eupatorin, resp.) showed that it possessed an inhibitory activity against *α*-glucosidase in normal rats loaded with starch and sucrose. The results showed that 1000 mg/kg of the 50% ethanolic extract of* O. stamineus* significantly (*P* < 0.05) decreased the plasma glucose levels of the experimental animals in a manner resembling the effect of acarbose. In streptozotocin-induced diabetic rats, only the group treated with 1000 mg/kg of the extract showed significantly (*P* < 0.05) lower plasma glucose levels after starch loading. Hence, *α*-glucosidase inhibition might be one of the mechanisms by which* O. stamineus* extract exerts its antidiabetic effect. Furthermore, our findings indicated that the 50% ethanolic extract of* O. stamineus* can be considered as a potential agent for the management of diabetes mellitus.

## 1. Introduction

Type 2 diabetes mellitus (DM) is a metabolic disease characterized by hyperglycemia, a condition which could either be attributed to insufficient insulin secretion or insulin resistance. The number of diabetic patients is rapidly rising in most parts of the world, especially in the developing countries such as Thailand, India, and Indonesia. Controlling blood glucose levels of diabetics within the normal range is mainly based on the use of oral hypoglycemic/antihyperglycemic agents and insulin. However, these conventional treatments have undesirable side effects [1–3]. Those shortcomings have led to a great interest in the use of medicinal plants as alternatives for the management of type 2 diabetes mellitus [4]. Control of postprandial plasma glucose levels is critical in the early treatment of diabetes mellitus and in reducing chronic vascular complications. Basically, a sudden rise in blood glucose levels, causing hyperglycemia in type 2 diabetic patients, would be due to starch hydrolysis by the *α*-amylase and *α*-glucosidases found in gastrointestinal tract [5]. Complex starches, oligosaccharides, and disaccharides must be broken down into monosaccharides (glucose and fructose) before they can be transported across the intestinal lumn (mainly in duodenum and upper jejunum) into the bloodstream and thereby increase blood glucose level. Thus, one of the effective strategies for the management of blood glucose level in type 2 DM is by inhibition of *α*-glucosidases and *α*-amylase [6, 7] which reduces the digestion of carbohydrates for production of monosaccharide and, hence, indirectly decreases blood glucose level. Among glucose lowering medications, *α*-glucosidase inhibitors delay the absorption of ingested carbohydrates, reducing the postprandial glucose and insulin peaks [8]. It was demonstrated that *α*-glucosidase inhibitors could be used to prevent disorders such as diabetes, obesity, hyperlipidaemia, and hyperlipoproteinaemia [9]. Our previous study showed that 50% ethanolic extract of* O. stamineus* and its active ingredient, sinensetin, were able to inhibit *α*-glucosidase and *α*-amylase* in vitro* [10]. To the best of our knowledge, there have been no other reports on* in vivo α*-glucosidase inhibitory activity of* O. stamineus*. The present study is an extension of our previous work, the objective of which is to conduct* in vivo α*-glucosidase inhibition studies on experimental animals to further understand the possible mechanisms of action by which the 50% ethanolic extract of* O. stamineus* exerts its antidiabetic effect.

## 2. Materials and Methods

### 2.1. Chemicals

Streptozotocin (Sigma Aldrich Chemical Co., USA), acarbose 50 mg (Bayer Pharmaceuticals, Leverkusen, Germany), starch (Ajax Chemicals, Sydney, Australia), sucrose and glucose (R & M Chemicals, Essex, UK), 3′hydroxy-5,6,7,4′-tetramethoxyflavone, sinensetin, and eupatorin (Indofine Chemical Company, New Jersey, USA) were purchased.

### 2.2. Plant Material and Extraction

Leaves of* Orthosiphon stamineus* were obtained from Kepala Batas, Pulau Pinang, Malaysia. The plant was identified at the School of Biological Sciences, Universiti Sains Malaysia, and a voucher specimen (10810) was deposited at its herbarium. The dried leaves were powdered using a milling machine and extracted with 50% (v/v) ethanol by maceration (200 g dried leaves in 2 L of 50% ethanol at 55°C for 24 hours, 2 cycles) over a period of 6 days. The extract was filtered and concentrated at 40°C using a rotary evaporator (Buchi Labortechnik, Flawil, Switzerland). Finally, the concentrated extract was freeze-dried (Labconco Corporation, Kansas City, MO, USA) to yield a 10.3% of dry powder.

### 2.3. HPLC Analysis of the Plant Extract

HPLC analysis was performed using a Shimadzu-LC system (Shimadzu, Japan) equipped with a CBM-20A controller, LC-20AT pump, DGU-20A5 degasser, SIL-20A autosampler, SPD-20AV detector, and CTO-10ASvp column oven.

Chromatographic separations were achieved using an Agilent Eclipse Plus C18 (250 × 4.6 mm i.d., 5 *μ*m). A Zorbax guard fittings kit packed with replaceable Eclipse Plus C18 Guard column (12.5 × 4.6 mm i.d., 5 *μ*m) was used to protect the analytical column. A reverse-phase HPLC assay was carried out using an isocratic system with a flow rate of 1 mL/min and a column temperature equaling 25°C. As the mobile phase, a mixture of acetonitrile, isopropyl alcohol, and 0.02 M phosphate buffer (NaH_2_PO_4_) (30 : 15 : 55 v/v) was used with the pH adjusted to 3.5 using 85% phosphoric acid. The UV detection was set at 340 nm. The injection volume was 20 *μ*L. Total run time was less than 20 min for each injection [11]. Data were acquired and processed with LC-Solution Software. The peaks were detected at 340 nm and identified using standard substances, namely, sinensetin, eupatorin, and 3′-hydroxy-5,6,7,4′-tetramethoxyflavone. The 50% ethanolic extract of* O. stamineus* prepared at 10 mg/mL served as the stock solution. To prepare the sample for injection, the stock solution was diluted with the mobile phase to a concentration of 1 mg/mL. The total amount of 3′hydroxy-5,6,7,4′-tetramethoxyflavone, sinensetin, and eupatorin in the 50% ethanol extract of* O. stamineus* was quantified using a developed HPLC method (*n* = 3). The quantities of these three compounds were then expressed as percentages of the dried extract.

### 2.4. Experimental Animals

Healthy adult male Sprague-Dawley rats weighing between 200 and 250 g were obtained from the Animal Research and Service Centre at Universiti Sains Malaysia (Penang, Malaysia) and housed in the Animal Transit Room at the School of Pharmaceutical Sciences, Universiti Sains Malaysia (Penang, Malaysia), five days prior to the experiment. All the animals used were approved by the Animal Ethics Committee, Universiti Sains Malaysia, and maintained in concordance with all international and national ethical guidelines. The animals had access to food and water* ad libitum*. Diabetes was induced in the animals by a single intraperitoneal injection of 60 mg/kg of streptozotocin in ice-cold citrate buffer, pH 4.5. Blood glucose levels were constantly monitored using an Accu-Chek Advantage-II Glucose meter (Roche Diagnostics, Manheim, Germany); and rats showing blood glucose levels approximately 15–17 mmol/L were included in the study. Acarbose was used as a positive control at a dose of 10 mg/kg.

### 2.5. Oral Carbohydrate Challenge Tests

The oral carbohydrate tolerance tests were conducted according to Ye et al. (2002) [12]. Basically, they were carried out using starch, sucrose, and glucose in normal and diabetic rats, respectively.

#### 2.5.1. Oral Starch Tolerance Test

Rats were divided into five groups consisting of six rats each (*n* = 6). The rats were fasted overnight for 12 h but had free access to water. The standardized 50% ethanol extract of* O. stamineus* was suspended in distilled water and administered oral doses of 250 mg/kg (group I), 500 mg/kg (group II), and 1000 mg/kg (group III). The control rats (group IV) received the vehicle (distilled water) only. Treatment (group V) rats were treated orally with acarbose at a dose of 10 mg/kg. Ten minutes thereafter, all the rats were loaded with starch orally at a dose of 3 g/kg. The tails were snipped for blood glucose estimation before (0 min) and at 30, 60, and 120 min after starch administration.

#### 2.5.2. Oral Sucrose Tolerance Test

The oral sucrose tolerance test was carried out similar to Section 2.5.1. However, instead of starch, the rats were loaded with sucrose at a dose of 4 g/kg.

#### 2.5.3. Oral Glucose Tolerance Test

The oral glucose tolerance test was carried out similar to Section 2.5.1. However, instead of starch, the rats were loaded with glucose at a dose of 2 g/kg.

### 2.6. Statistical Analysis

Data were expressed as mean ± standard error of mean (SEM); and the statistical analysis was performed using one-way analysis of variance (ANOVA). Significant differences between the control and the experimental groups were determined using the LSD multiple comparison test. Differences with *P* < 0.05 were considered to be significant.

## 3. Results

### 3.1. Phytochemical Composition

**Figure 1:**
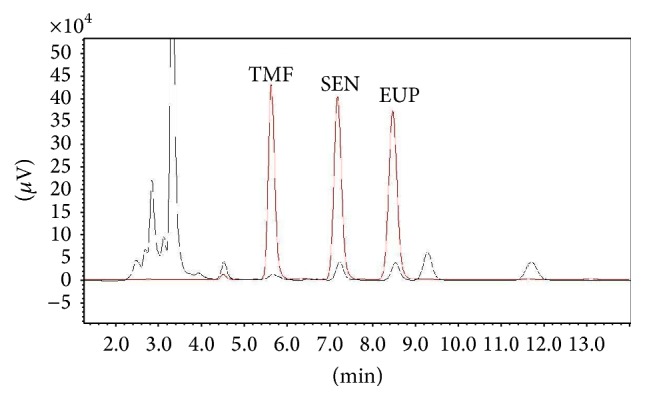
HPLC chromatogram of standard markers and 50% ethanolic extract of* O. stamineus*. Standard compounds: TMF (3′-hydroxy-5,6,7,4′-tetramethoxyflavone), SEN (sinensetin), and EUP (eupatorin) (red chromatogram). HPLC chromatogram of 50% ethanolic extract of* O. stamineus* (black chromatogram).

HPLC analysis showed the percentages of 3′hydroxy-5,6,7,4′-tetramethoxyflavone, sinensetin, and eupatorin in the 50% ethanol extract of* O. stamineus* to be 1.02 ± 0.0007, 3.76 ± 0.02, and 3.032 ± 0.03, respectively (Figure 1).

### 3.2. Oral Starch Tolerance Test

**Figure 2:**
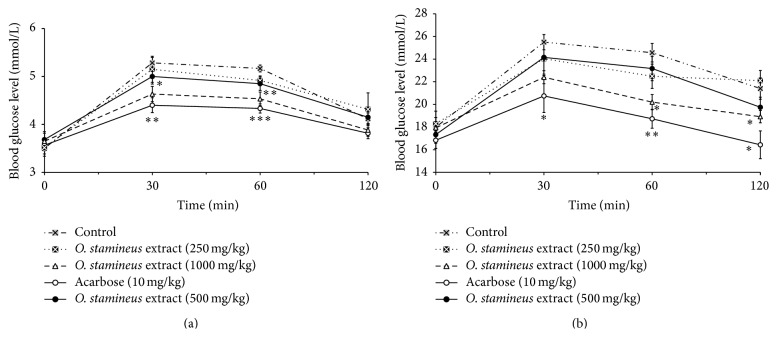
The effect of oral administration of 50% ethanol extract of* O. stamineus* at doses of 250, 500, and 1000 mg/kg on blood glucose levels of normal rats (a) and diabetic rats (b) loaded with 3 g/kg of starch (a). Values are expressed as mean ± SEM (*n* = 6); ^*∗*^ *P* < 0.05 and ^*∗*^ *P* < 0.01 compared with the control.

Using normal rats, extract-treated groups receiving 250 mg/kg and 500 mg/kg of the 50% ethanol extract of* O. stamineus* did not show any significant decrease in blood glucose levels (Figure 2(a)) compared to control rats. Only a dose of 1000 mg/kg of the extract reduced the blood glucose levels significantly (*P* < 0.05) after starch loading. Administered at a dose of 10 mg/kg, acarbose also significantly (*P* < 0.05) reduced the blood glucose levels of starch loaded normal rats. Likewise, using diabetic rats, only the group treated with 1000 mg/kg showed significant (*P* < 0.05) decrease in blood glucose levels following starch loading (Figure 2(b)). As in the normal rats, 10 mg/kg of acarbose also significantly (*P* < 0.05) decreased the blood glucose levels of the diabetic rats.

### 3.3. Oral Sucrose Tolerance Test

**Figure 3:**
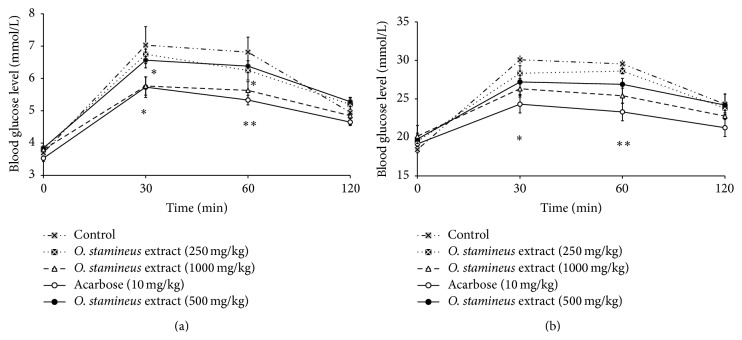
The effect of oral administration of 50% ethanol extract of* O. stamineus* at doses of 250, 500, and 1000 mg/kg on blood glucose levels of normal rats (a) and diabetic rats (b) loaded with 4 g/kg of sucrose. Values are expressed as mean ± SEM (*n* = 6); ^*∗*^ *P* < 0.05 and ^*∗*^ *P* < 0.01 compared with the control.

In normal rats, 250 mg/kg and 500 mg/kg of the 50% ethanol extract of* O. stamineus* did not show any reduction in blood glucose levels after sucrose loading. However, 1000 mg/kg of the extract managed to lower the blood glucose levels significantly (*P* < 0.05) compared to the control group (Figure 3(a)). Acarbose given at a dose of 10 mg/kg also produced a significant (*P* < 0.05) blood glucose lowering response in the sucrose-loaded normal rats. In diabetic rats, none of the extract-treated groups (250 mg/kg, 500 mg/kg, and 1000 mg/kg) showed any significant decrease in the blood glucose levels as compared to the diabetic control group (Figure 3(b)), whereas acarbose produced a significant (*P* < 0.05) blood glucose lowering effect in the sucrose-loaded STZ-induced diabetic rats.

### 3.4. Oral Glucose Tolerance Test

**Figure 4:**
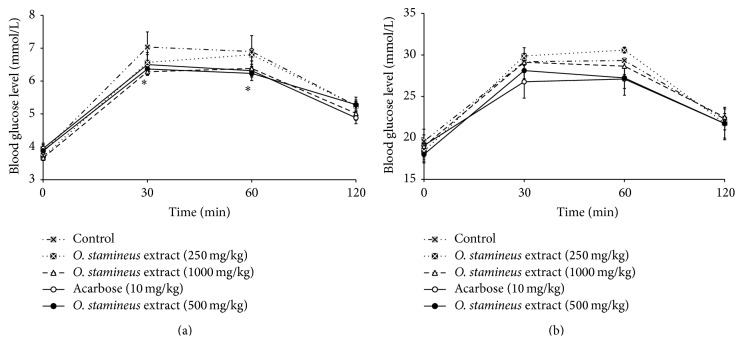
The effect of oral administration of 50% ethanol extract of* O. stamineus* at doses of 250, 500, and 1000 mg/kg on blood glucose levels of normal rats (a) and diabetic rats (b) loaded with 2 g/kg of glucose. Values are expressed as mean ± SEM (*n* = 6); ^*∗*^ *P* < 0.05 compared with the control.

In normal rats, none of the groups treated with the 50% ethanol extract of* O. stamineus* showed any significant decreases in the blood glucose levels. Only acarbose produced a significant (*P* < 0.05) blood glucose lowering effect after glucose loading (Figure 4(a)). Similarly, using diabetic rats, none of the administered doses of the extract caused significant reduction in the blood glucose levels of glucose-loaded STZ-induced diabetic rats (Figure 4(b)). The acarbose-treated diabetic rats also did not show any significant reduction in the blood glucose levels.

## 4. Discussion

Diabetes mellitus is a metabolic disorder of multiple etiologies, characterized by chronic hyperglycemia with disturbances in carbohydrate, fat, and protein metabolism, resulting from defects in insulin secretion, insulin action, or both [13]. One therapeutic approach for treating diabetes is to decrease postprandial hyperglycemia. This is done by delaying the absorption of glucose by inhibiting the carbohydrate hydrolyzing enzymes, *α*-amylase and *α*-glucosidase in the digestive tract. Inhibitors of these enzymes delay carbohydrate digestion and prolong its overall digestion time, causing a reduction in the rate of glucose absorption and consequently blunting the postprandial increase in plasma glucose [14]. Examples of such inhibitors in clinical use are acarbose, miglitol, and voglibose [15]. The reduction in the postprandial blood glucose levels caused by *α*-glucosidase inhibitors, such as miglitol and acarbose, following a starch load is well established. These agents act by inhibiting the last step in carbohydrate digestion, namely, the conversion of disaccharides to monosaccharides (glucose), resulting in a consequent decrease in the rate of entry of glucose into the systemic circulation.

Several classes of chemicals have been found in* O. stamineus*, proving it to be rich in flavonoids, terpenoids, caffeic acid derivatives, chromene, and phenolic compounds [16–18]. Some of the present phenolic compounds and flavonoids possess marked antidiabetic activities. Moreover,* O. stamineus* aqueous extract has been proven to exert antidiabetic and lipid lowering effects in diabetic rats [4]. Although there have been reports on the antihyperglycemic [4] and antidiabetic [19] activities of* O. stamineus* aqueous extracts, attributing their activities to free radical scavenging and, in part, to increased glucose metabolism, there have been no previous reports, at least to the best of our knowledge, on the* in vivo* activity of this plant extracts in relation to *α*-glucosidase and *α*-amylase inhibition.

This was the first study to evaluate the* in vivo α*-glucosidase inhibitory effect of* O. stamineus* extract in normal and STZ-induced diabetic rats. In the oral carbohydrate challenge tests, doses of 250 mg/kg and 500 mg/kg of a 50% ethanolic extract of* O. stamineus* did not reduce blood glucose levels after oral starch or sucrose loading either in normal or diabetic rats. The highest dose of 1000 mg/kg of the 50% ethanolic extract of* O. stamineus* reduced blood glucose levels after starch loading in both normal and diabetic rats. However, upon sucrose loading, the 1000 mg/kg dose only caused a blood glucose lowering response in normal rats but did not do so in diabetic animals. In the oral glucose tolerance test, 1000 mg/kg of the extract failed to show any significant blood glucose lowering activity either in normal or diabetic rats. Nevertheless, the above results for the 1000 mg/kg dose showed a striking similarity to the effects of acarbose. These findings also agreed with the previous studies in which 50% ethanolic extract of* O. stamineus* and the isolated sinensetin compound showed inhibitory activity on *α*-glucosidase and *α*-amylase* in vitro* [10]. Acarbose caused a significant reduction in blood glucose levels upon starch and sucrose loading in normal and diabetic rats. In contrast, it failed to inhibit the increase in the blood glucose levels of glucose-loaded diabetic rats. *α*-Glucosidase inhibitors are competitive inhibitors of the small intestinal *α*-glucosidase enzymes that break down nonabsorbable oligosaccharides [20]. The most commonly used *α*-glucosidase inhibitor of present so far is acarbose. Chemically, acarbose is an oligosaccharide produced by cultured strains of actinomycetes. It is a competitive inhibitor with a higher affinity for sucrose than glucoamylase and pancreatic *α*-amylase [21]. The 50% ethanolic extract of* O. stamineus* seems to delay the rapid digestion of starch and sucrose, thus, lengthening the time needed for carbohydrate absorption. It may be that sinensetin or some other unknown compounds in the extract are responsible for this reduction in the blood glucose levels of the normal rats. The tendency of the 50% ethanolic extract of* O. stamineus* to suppress the increase in blood glucose levels of starch loaded diabetic rats suggests the involvement of *α*-glucosidase inhibition. Acarbose-like drugs that inhibit the *α*-glucosidases present in the epithelium of the small intestine, have been shown to decrease postprandial hyperglycemia [22] and to improve impaired glucose metabolism without promoting insulin secretion in noninsulin dependent diabetes mellitus (NIDMM) patients [23]. These medications should be most useful for people who have just been diagnosed with type 2 diabetes and who have blood glucose levels that are only slightly above the level considered to be alarming for a diabetic. They are also useful for people who take a sulfonylurea (e.g., glibenclamide) or a biguanide (e.g., metformin) derivative as a sole medication and who would need additional medications to keep their blood glucose levels within a safe range. Therefore, delaying carbohydrate absorption with a plant-based *α*-glucosidase inhibitor such as* O. stamineus* extract offers a prospective therapeutic approach for the management of type 2 diabetes mellitus and may be beneficial for borderline diabetic patients [24].

Although this extract seems to be promising in the treatment of type 2 diabetes mellitus by reducing postprandial hyperglycemia, it is still too early to recommend its use in human. Only a thorough and full-fledged study could rationalize its use in human. The results suggest that the *α*-glucosidase inhibitory effect of the 50% ethanolic extract of* O. stamineus* may contribute to delaying carbohydrate digestion. Therefore, *α*-glucosidase inhibition could possibly be one of the mechanisms by which the 50% ethanolic extract of* O. stamineus* exerts its antidiabetic effects—indicating that* O. stamineus* could be considered as a potential drug in the management of NIDDM. The present results showed that* O. stamineus* extract significantly reduced the plasma glucose concentrations of sucrose-loaded normal and diabetic rats. Thus,* O. stamineus* extract may be beneficial for patients with diabetes mellitus.
